# The efficacy of neurotropin in treating patients with nummular headaches

**DOI:** 10.1186/1129-2377-14-S1-P40

**Published:** 2013-02-21

**Authors:** D Danno, H Tachibana

**Affiliations:** 1 Hyogo College of Medicine, Japan

## Introduction

A nummular headache is a headache that is characterized by pain localized to a small, circumscribed area of the head, typically measuring 2 to 6 cm in diameter in the absence of any lesion of the underlying structures.

## Purpose

Neurotropin is a non-protein extract isolated from the inflamed skin of rabbits inoculated with the vaccinia virus that is used to treat neuropathic pain. In this study, we reviewed three cases retrospectively to assess the possible efficacy of neurotropin in patients with nummular headaches.

## Methods

Three nummular headache patients participated in this study. For each patient, the diagnosis of nummular headache was made based on the ICHD - II nummular headache diagnostic criteria. We prescribed neurotropin at a dose of 16 NU/day to the patients during the clinical course of this study.

## Results

Case 1: A 39-year-old male developed bifocal pain one week previously in a circumscribed area of the left temporal region measuring 4 cm in diameter. Treatment with NSAIDs was determined to be invalid. Treatment with neurotropin relieved the patient's pain, and the headache resolved after four days. Case 2: A 57-year-old female developed bifocal pain one week previously in a circumscribed area including both temporal regions measuring 3 cm in diameter. Treatment with neurotropin relieved the patient's pain slightly; however, the headache remained. Case 3: A 71-year-old male developed pain more than one year previously in a circumscribed area of the left parietal region measuring 3 cm in diameter. Treatment with NSAIDs was determined to be invalid. Treatment with neurotropin relieved the patient's pain, and the headache resolved after three months. In two of the three cases of patients with nummular headaches, treatment with neurotropin was found to be very effective for pain reduction.

## Conclusion

The present results suggest that prescribing neurotropin may be a possible choice for treating patients with nummular headaches.
